# Abnormal activity in the brainstem affects gait in a neuromusculoskeletal model

**DOI:** 10.1186/s12984-025-01596-x

**Published:** 2025-04-04

**Authors:** Daisuke Ichimura, Makoto Sawada, Kenji Wada, Ritsuko Hanajima

**Affiliations:** 1 Artificial Intelligence Research Center, National Institute of Advanced Industrial Science and Technology, Tokyo, Japan; 2 School of Physical Therapy, Faculty of Rehabilitation, Reiwa Health Sciences University, Fukuoka, Japan; 3 Department of Dementia Medicine, Kawasaki Medical School, Okayama, Japan; 4 Department of Brain and Neurosciences, Faculty of Medicine, Tottori University, Tottori, Japan

**Keywords:** Pathological locomotion, Neuromusculoskeletal model, Brainstem, Freezing of gait, Central pattern generator, Midbrain locomotor region, Parkinson’s disease

## Abstract

**Background:**

The ability to start and stop locomotion in response to different situations is an essential survival strategy in mammals. Mammalian locomotion is controlled by central pattern generators in the spinal cord, which are modulated by higher centers, particularly by the stimulation of the midbrain locomotor region. The midbrain locomotor region consists of the pedunculopontine nucleus and cuneiform nucleus, each having different roles in animals. Optogenetic activation of the cuneiform nucleus increases locomotion activities, whereas that of pedunculopontine nucleus decreases them. In neurological disorders such as Parkinson’s disease, patients exhibit disturbed locomotion controls, including freezing of gait, which is defined as “a brief, episodic absence or marked reduction in the forward progression of the feet despite the intention to walk.” However, the details and pathophysiological mechanisms of freezing of gait remain unclear.

**Methods:**

In this study, we aimed to elucidate the mechanisms underlying freezing of gait using a two-dimensional neuromusculoskeletal model fixed on the sagittal plane. This model consisted of a body with seven links and 18 muscles as well as a neural system including the brainstem and spinal cord. We developed a normal condition model and then derived a model of abnormal brainstem activity by modifying the parameters of the pedunculopontine nucleus and cuneiform nucleus during the initial 3 s of walking.

**Results:**

The normal models walked successfully following internal parameter optimization using standard genetic algorithms. In an abnormal model, 156 freezing of gait events were detected among 40,000 parameter sets using a freezing of gait-identifying algorithm. Hierarchical cluster analysis identified four clusters of parameters, based on the intensities of the pedunculopontine nucleus and cuneiform nucleus activity, differentiated in physiological movement types during freezing of gait events that were similar to the clinical classification types of freezing of gait.

**Conclusions:**

Our results indicate that pedunculopontine nucleus and cuneiform nucleus activities could be linked with freezing of gait and that different modifications of those activities could generate observed freezing of gait subtypes. Our models can provide insights relevant for understanding the pathophysiological mechanisms of freezing of gait and are expected to assist in the classification of freezing of gait subtypes.

**Supplementary Information:**

The online version contains supplementary material available at 10.1186/s12984-025-01596-x.

## Background

The ability to start and stop locomotion in response to different situations is an essential survival strategy in mammals, including humans. Mammalian locomotion is controlled by central pattern generators (CPGs) in the spinal cord, which generate the basic rhythm of gait and coordinate the flexor and extensor muscles through motor neuron activity [1, 2]. CPGs are modulated by higher centers, particularly by the stimulation of the midbrain locomotor region (MLR), which allows the initiation and termination of locomotion [3, 4].

In decerebrated cats, wherein spinal cord connections are disconnected from the cerebrum, locomotion can be induced by stimulating the MLR [4], which indicates that the MLR is responsible for locomotion. The MLR consists of the pedunculopontine nucleus (PPN) and cuneiform nucleus (CnF), each having different roles in animals [5–11]. In studies using the optogenetic activation of MLR glutamatergic neurons in mice, locomotor activity increased with CnF stimulation and decreased with PPN stimulation [7–9]. However, the effect of MLR activity on bipedal locomotion in humans remain unclear [12]. Although functional abnormalities in MLR activity have been identified in the pathological gait related to walking initiation, bipedal locomotion is difficult to validate using data from quadrupedal rats.

Freezing of gait (FOG) is defined as “a brief, episodic absence or marked reduction in the forward progression of the feet despite the intention to walk” [13]. It is common in Parkinson’s disease (PD) or progressive supranuclear palsy, inducing falls and injuries [14, 15]. To prevent such incidents, several methods have been proposed for identifying FOG in daily life, including the use of wearable devices [16, 17]. However, because the direct investigation of areas responsible for bipedal locomotion in humans, such as the brainstem and spinal cord, is technically and ethically difficult, the pathogenesis of FOG remains unclear. Moreover, therapeutic medications have not been consistently effective in FOG treatment [18], and several clinical subtypes exist (complete akinesia, shuffling with small steps, and trembling in place) [19, 20], which complicates the development of tailored treatments for this condition. Therefore, clarifying FOG pathogenesis may help identify rehabilitation methods tailored to each severity and subtype, medication adjustments, and might aid the development of effective devices.

Several neuromusculoskeletal models of bipedal locomotion have been used to investigate the biomechanics and motor control of human gait [21–26]. The forward dynamics simulation technique allows the generation of various modeled physical and neural changes, offering a “what if” approach with great potential for the causal investigation of pathological conditions [27–30].

The aim of this study was to computationally elucidate the pathogenesis of FOG and its subtypes using a two-dimensional neuromusculoskeletal model. We hypothesized that by modifying the CnF and PPN activity parameters in the MLR model, FOG would be observed and its classification would resemble the clinical subtypes. In particular, we verified the primary factors of FOG by modeling the MLR in the brainstem and CPG in the spinal cord and by simulating gait using the lowest possible number of components. We investigated whether FOG could be observed during the initial 3 s of walking by modifying the parameters of the CnF and PPN neurons in the MLR model. We identified FOGs occurring based on 40,000 parameter sets of PPN and CnF neurons using an FOG-identifying algorithm. The identified FOGs were then grouped using hierarchical cluster analysis (HCA) for comparison with the qualitative FOG classification used in clinical practice. We confirmed that the resulting classification of FOG resembled clinical findings by modifying the activities in the MLR model. Our findings provide insights into FOG pathogenesis and lay a strong foundation for future clinical research in this field.

## Methods

### Musculoskeletal model

**Fig. 1:**
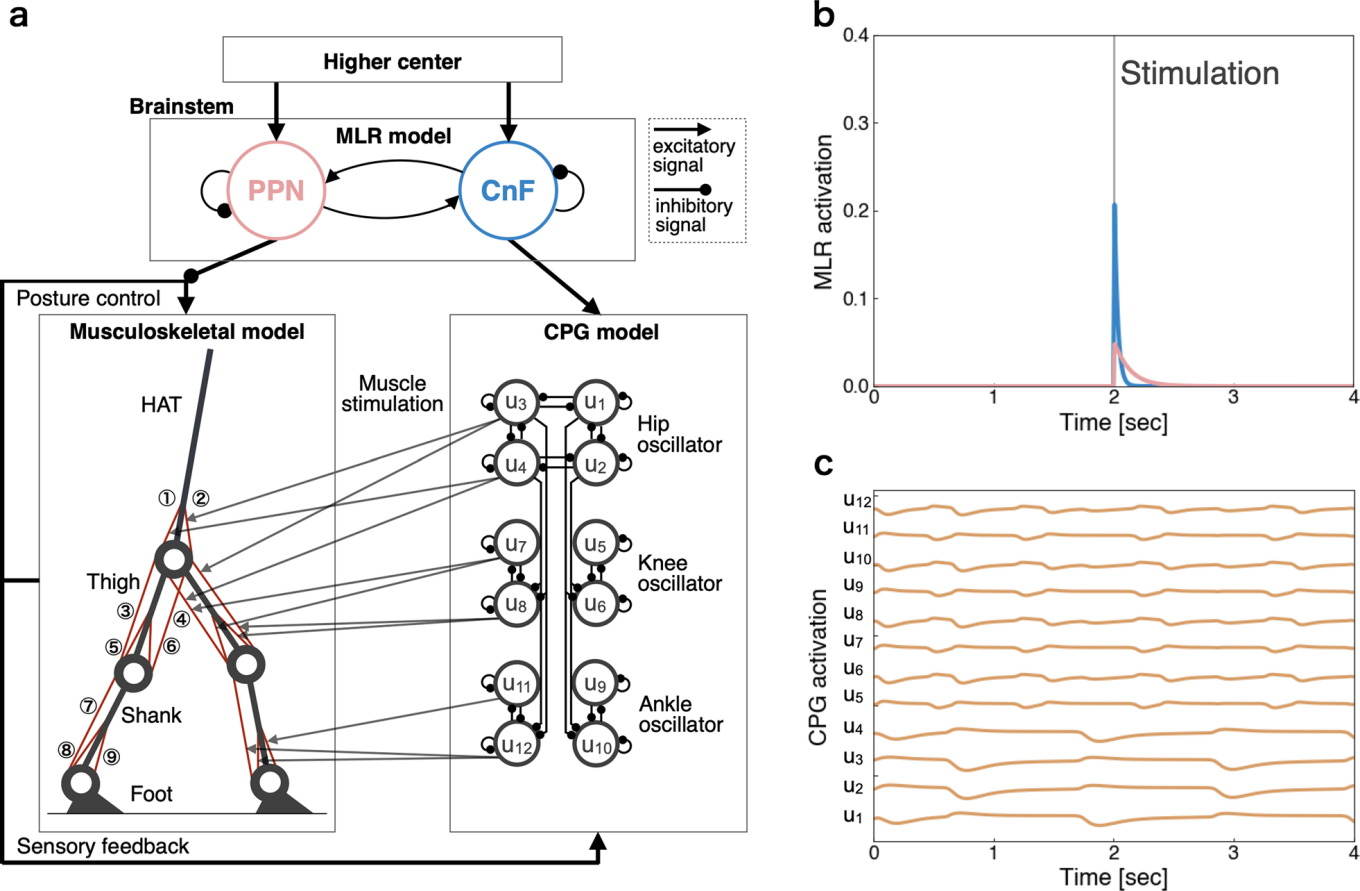
Schematic of the neuromusculoskeletal model. **a** The model consisted of the midbrain locomotor region (MLR) in the brainstem, central pattern generators (CPGs) in the spinal cord, and a musculoskeletal model. The MLR consisted of the pedunculopontine nucleus (PPN) and cuneiform nucleus (CnF), with their activities modulated by excitatory signals from the higher center. The PPN modulated posture control in the musculoskeletal model with inhibitory signals, and the CnF modulated activity in the CPG model with excitatory signals. The musculoskeletal model consisted of seven links representing the HAT (head, arms, and torso), thighs, shanks, and feet, and the muscles consisted of (1) gluteus maximus (GM), (2) iliopsoas (IL), (3) biceps femoris long head (BFL), (4) rectus femoris (RF), (5) biceps femoris short head (BFS), (6) vastus (VA), (7) gastrocnemius (GC), (8) soleus (SO), and (9) tibialis anterior (TA). The CPG model consisted of 12 internal units (*u*_1_, …, *u*_12_) generating hip, knee, and ankle oscillators. The output of the CPG model corresponded to each muscle model. **b** PPN and CnF activities in response to stimulation (gray line) over 0.01 s at 2 s. PPN showed long-lasting activity, whereas CnF showed short-lasting activity, as reported by Dautan et al. [9]. Red and blue indicate PPN and CnF activities, respectively. **c** CPG activity without feedback. Each neuron exhibited periodic activity

We developed a two-dimensional musculoskeletal model that included the head, arms, torso (HAT), thighs, shanks, and feet (Fig. 1). Segment size and inertia parameters were set as described by Jo and Massaquoi, Ichimura and Yamazaki, and Ichimura et al. [24, 28, 30] (see Additional file 1). All joints were modeled as pin joints and had a linear viscous component. The hip, knee, and ankle joints had viscosity coefficients of 1.09, 3.17, and 0.943 Nms rad^−1^, respectively [25, 28, 30]. The angles of the knee and ankle joints have limited ranges of motion, from − 2.8 to − 0.1 rad and − 1.0 to 0.54 rad, respectively. When these joint angles are beyond their limits, they are subject to linear elastic and damping torque. The elastic and viscous coefficients were 2.0 × 10^3^ Nm rad^−1^ and 3.0 × 10^2^ Nms rad^−1^ for the knee joint and 2.0 × 10^3^ Nm rad^−1^ and 3.0 × 10 Nms rad^−1^ for the ankle joint [25]. When the heels or toes contact the ground, they receive the ground reaction forces generated by the spring and damper. The coefficients of the springs and dampers were 5.0 × 10^3^ N m^−1^ and 1.0 × 10^2^ Ns m^−1^ in the horizontal direction and 2.5 × 10^4^ N m^−1^ and 1.0 × 10^3^ Ns m^−1^ in the vertical direction, respectively. Nine primary muscles were placed in each leg (Fig. 1): the gluteus maximus, iliopsoas (IL), biceps femoris long head, rectus femoris, biceps femoris short (BFS), vastus, gastrocnemius, soleus, and tibialis anterior. When muscles receive signals from the corresponding α-motoneurons, they generate muscle tension through force–length and force–velocity relationships. We used the following muscle model [22, 25, 30]:

1 $$\begin{aligned}{F}_{m} &={\overline{F} }_{m}^{\text{CE}}\cdot k\left({\xi }_{m}\right)\cdot h\left({\eta }_{m}\right)\cdot {\alpha }_{m}+{F}_{m}^{\text{PD}}+{F}_{m}^{\text{PE}}, \\ k\left({\xi }_{m}\right)&=0.32+0.71{\text{exp}}\left(-1.112\left({\xi }_{m}-1.0\right)\right){\text{sin}}\left(3.722\left({\xi }_{m}-0.656\right)\right),\\ h\left({\eta }_{m}\right)&=1+{\text{tanh}}\left(3.0{\eta }_{m}\right),\\ {F}_{m}^{\text{PD}}&={c}_{m}^{\text{PD}}{\dot{L}}_{m}, \\ {F}_{m}^{\text{PE}}&={k}_{m}^{\text{PE}}({\text{exp}}\left(15\left({L}_{m}-{\overline{L} }_{m}\right)\right)-1.0), \end{aligned}$$

where ${F}_{m}$ is the muscle tension produced by the *m-*th muscle, ${\overline{F} }_{m}^{\text{CE}}$ is the maximum muscle tension, $k\left({\xi }_{m}\right)$ is the force–length relationship, $h\left({\eta }_{m}\right)$ is the force–velocity relationship, ${\alpha }_{m}$ is the stimulus signal from the corresponding α-motor neuron (0 ≤ ${\alpha }_{m}$  ≤ 1), ${F}_{m}^{\text{PD}}$ and ${F}_{m}^{\text{PE}}$ are the forces generated by the damping and elastic elements, respectively. ${\xi }_{m}$ and ${\eta }_{m}$ are the normalized muscle length and contraction velocity divided by the muscle optimum length ${\overline{L} }_{m}$ and the muscle maximum contraction velocity ${\overline{\dot{L}} }_{m}$, respectively. Thus, ${\upxi }_{m}={L}_{m}/{\overline{L} }_{m}, {\upeta }_{m}={\dot{L}}_{m}/{\overline{\dot{L}} }_{m}$, and ${\overline{\dot{L}} }_{m}$ are the muscle length and contraction velocity, respectively. ${c}_{m}^{\text{PD}}$ is the viscosity coefficient, and ${k}_{m}^{\text{PE}}$ is the modulus of the elastic elements. These parameters were previously used by Aoi et al. and Ogihara and Yamazaki [22, 25] and were determined based on anatomical drawings and the models proposed by Davy and Audu [31] (see Additional file 1).

### Nervous system model

Biological experiments have shown that CnF and PPN anatomically connect with each other and with inputs from different brain regions, respectively [7, 9]. We, therefore, constructed an MLR model consisting of PPN and CnF with such connections. This model was based on the Matsuoka model [32], which is widely used as a neural rate model [21–23, 30, 33]. To investigate locomotion control by MLR in the brainstem and CPG in the spinal cord, we modeled them computationally. The MLR model consists of the PPN and CnF, which are described by the following equations:

2 $$\begin{array}{c}\begin{array}{c}{\tau }^{\text{PPN}}{\dot{u}}^{\text{PPN}}=-{u}^{\text{PPN}}+{w}^{{\text{PPN}}\leftarrow {\text{CnF}}}\text{max(0, }{u}^{\text{CnF}}\text{)}-{\beta }^{\text{PPN}}{u}^{\text{PPN}}+{{w}^{\text{HC}}{\text{HC}}}^{\text{PPN}}(t),\\ {\tau }^{\text{CnF}}{\dot{u}}^{\text{CnF}}=-{u}^{\text{CnF}}+{w}^{{\text{CnF}}\leftarrow {\text{PPN}}}\text{max(0, }{u}^{\text{PPN}}\text{)}-{\beta }^{\text{CnF}}{u}^{\text{CnF}}+{{w}^{\text{HC}}{\text{HC}}}^{\text{CnF}}(t),\\ {\text{HC}}^{\text{PPN}}\left(t\right)=\left\{\begin{array}{c}{s}^{\text{PPN}} \left(0.0\le t\le 3.0\right)\\ 1.0 \left(3.0<t\right)\end{array}\right., \\ {\text{HC}}^{\text{CnF}}(t)=\left\{\begin{array}{c}{s}^{\text{CnF}} (0.0\le t\le 3.0)\\ 1.0 \ (3.0<t)\end{array}\right., \end{array}\end{array}$$

where ${u}^{\text{PPN}}$ and ${u}^{\text{CnF}}$ are variables representing the internal states of PPN and CnF neurons, respectively; ${\tau }^{\text{PPN}}$ and ${\tau }^{\text{CnF}}$ are time constants; and ${\beta }^{\text{PPN}}$, ${\beta }^{\text{CnF}}$, and ${w}^{\text{HC}}$ are coefficients. ${w}^{{\text{PPN}}\leftarrow {\text{CnF}}}$ is the connection weight from the CnF neuron to the PPN neuron and, ${w}^{{\text{CnF}}\leftarrow {\text{PPN}}}$ is the connection weight from the PPN neuron to the CnF neuron. ${\text{HC}}^{\text{PPN}}(t)$ and ${\text{HC}}^{\text{CnF}}(t)$ indicate external inputs from a higher center. ${s}^{\text{PPN}}$ and ${s}^{\text{CnF}}$ indicate activity intensities of PPN and CnF neurons, respectively. Based on previous electrophysiological studies [7, 9], the input from CnF to PPN was larger (${w}^{{\text{PPN}}\leftarrow {\text{CnF}}}=0.10$) than the input from PPN to CnF (${w}^{{\text{CnF}}\leftarrow {\text{PPN}}}=0.01$), and the time constants were set to relative values such that PPN was larger than CnF. An additional text file presents the other parameter values (see Additional file 1). We used the following mathematical model for the spinal cord that produces the gait rhythm as a CPG [21]:

3 $$\begin{array}{c}{\tau }_{i}\dot{{u}_{i}}=-{u}_{i}+{\sum }_{j=1}^{12}{w}_{i j}^{\text{CPG}}{ y}_{j}-\beta {v}_{i}+{u}_{0}{u}^{\text{CnF}}+{\text{Feed}}_{i}\left(\{{\theta }_{l}^{\text{seg}}{\}}_{l},\{\text{GRF}_{s}{\}}_{s} | \{{w}_{k}^{\text{Feed}}{\}}_{k}\right),\\ {\tau }_{i}^{\prime}{\dot{v}}_{i}=-{v}_{i}+{y}_{i},\\ {y}_{i}={\text{max}}\left(0,{u}_{i}\right),\end{array}$$

where ${u}_{i}$ is the internal state of the *i-*th neuron and ${v}_{i}$ is a variable representing the self-inhibitory effect of the *i-*th neuron. ${\tau }_{i}$ and ${\tau }_{i}^{\prime}$ are time constants, $\beta$ is a coefficient, and ${w}_{i j}^{\text{CPG}}$ is a connecting weight from the *j-*th neuron to the *i-*th neuron. ${u}_{0}$ is the external input weight of the CnF neuron. ${\text{Feed}}_{i}$ is a feedback signal from the musculoskeletal system. ${\theta }_{l}^{\text{seg}}$ is the segment angle (*l* $\in \{{\text{HAT}}, {\text{thigh}}, {\text{shank}}, {\text{foot}} \}$ for each leg), GRFs is the vertical ground reaction force ($s\in \{\text{left limb}, \text{right limb}\}$), and ${w}_{k}^{\text{Feed}}$ is the weight coefficient ($k = 1, \dots , 16$). Parameter values are as shown in an additional text file (see Additional file 1). ${y}_{i}$ excites α-motor neurons, which in turn activate the muscles. The α-motor neurons also receive feedback signals from various reflexes, such as the postural control and the cross-stretch reflex. α-Motor neuron output ${\alpha }_{m}$ and reflex output were calculated as follows:

4 $$\begin{array}{c}{\alpha }_{m}=\frac{2.0}{1.0+{\text{exp}}\left(0.25\left({\sum }_{i=1}^{18}{w}_{m i }^{\alpha }{y}_{i}+\frac{1.0}{{u}^{\text{PPN}}}{\text{P}}_{m}\left(\{{\theta }_{j}{\}}_{j},\{{\theta }_{l}^{\text{seg}}{\}}_{l},\{{\text{GRF}}_{s}{\}}_{s} | \{{w}_{o}^{\text{POS}}{\}}_{o}\right)\right)\right)}-1.0,\ \end{array}$$

where ${w}_{m i}^{\alpha }$ and ${w}_{o}^{\text{POS}}$ are weight coefficients ($o = 1, \dots , 23$), ${\text{P}}_{m}$ is posture control affected by PPN neurons, and ${\theta }_{j}$ is the joint angle (*j* $\in \{{\text{hip}}, {\text{knee}}, {\text{ankle}}\}$). The parameter values and physical responses in the posture controls are listed in an additional text file (see Additional file 1).

Thus, increased ${u}^{\text{PPN}}$ in the PPN model decreased postural control and increased ${u}^{\text{CnS}}$ in the CnF model increased activity in the CPG model. These activity alterations assumed that PPN stimulation decreases muscle tone and that CnF stimulation increases locomotion [6, 9].

### Generation of normal and abnormal locomotion

Our model features 49 free parameters (${u}_{0}$, ${w}_{k}^{\text{Feed}}$, ${w}_{i j}^{\text{CPG}}$, and ${w}_{o}^{\text{POS}}$) that need to be adjusted to generate adequate behaviors. These parameters were optimized to acquire bipedal locomotion using a standard genetic algorithm (GA) [22, 23, 30]. First, the free parameters were optimized using the GA with a message-passing interface, which is a library for parallel computing, to simulate a normal gait. We employed the evaluation function *J* to maximize, which is given by the following equation:

5 $$\begin{array}{c}\begin{array}{c}J=\left\{\begin{array}{c}1.5D+0.5S+0.1T+P+5.0 \left(D<10{\text{m}}\right),\\ 1.0D+0.2S+P+\frac{25}{C}+20.0 \left(D\ge 10{\text{m}}\right),\end{array}\right.\\ C=\frac{1.0}{\textit{TMV}}{\int }_{t=0}^{T}{\sum }_{m=1}^{18}{\dot{E}}_{m}dt,\end{array}\ \end{array}\begin{array}{c}\ \end{array}$$

where $D$ is the distance walked until the model falls, $S$ is the number of steps, ${\textit{T}}$ is the locomotion duration, $P$ =  − 2.5 is the penalty applied when the model falls, and $C$ is the gross metabolic cost of transport [34]. *M* and *V* represent body mass, and walking speed, respectively. ${\dot{E}}_{m}$ is the metabolic energy consumption of all muscles [35]. All programmes were written in C language, and the fourth-order Runge–Kutta method was used for the numerical solution of the differential equations. The time-step size was set to 0.1 ms. We performed five simulations with five different random number generator seeds in the normal model and confirmed that the simulation results were uniquely determined. We observed stable bipedal locomotion for 15 s, with no qualitative differences in locomotion patterns owing to differences in seeds.

We then modified the parameters of the PPN and CnF models to set gait initiation difficulty in the FOG. Based on a previous report that 70% of FOGs last less than 5 s [17], the PPN and CnF neurons changed only during the initial 3 s of walking (Eq. 2). For a thorough investigation, the values of ${s}^{\text{PPN}}$ and ${s}^{\text{CnF}}$ were permutatively changed from 0.00 to 2.00 in increments of 0.01 (40,000 values).

### Data processing

After gait simulations with 40,000 different parameter sets of PPN and CnF neurons, we detected the FOG from the simulation results using an FOG-identifying algorithm that is clinically used [17]. This open-source algorithm was used to analyze the correlation of angular velocity between the right and left lower legs and the freezing ratio calculated from the acceleration of the lower leg. The freezing ratio was defined as the power in the freezing band (3–10 Hz) divided by the power in the locomotor band (0–3 Hz) using the Fast Fourier Transform method, with a larger ratio indicating greater freezing. If the correlation between the left and right leg was low and the freezing ratio was high, this algorithm declared a FOG episode. In clinical practice, wearable inertial sensors allow the implementation of this algorithm. The area under curve (AUC) value for FOG identification was approximately 0.9 versus clinical raters, which was highly functional.

The detected FOG simulations were analyzed using HCA [36], which identified clusters based on two variables, ${\text{s}}^{\text{PPN}}$ and ${\text{s}}^{\text{CnF}}$. Euclidean distance was selected as the metric, and Ward’s linkage method was employed for this analysis [37–39]. Individual clusters were serially combined in the HCA to form new clusters. This process ended by grouping all trials into a single cluster that formed a hierarchical tree (dendrogram). The final number of clusters was decided by the agglomeration coefficient while increasing the number of clusters and employing a stopping rule (a large percentage increase in the coefficient decrease followed by a plateau) [36–39]. The number of clusters was also verified by visual inspection of the dendrogram.

### Statistical analysis

After forming clusters, one-way analysis of variance was performed for leg motion and effective forward motion based on the qualitative FOG classification used in clinical practice [19, 20]. The leg motion and effective forward motion corresponded to the freezing ratio and walking distance, respectively, during the first 3 s of walking. The normality of variables was tested using the Shapiro–Wilk test, and the equality of variances was tested using the Levene’s test. When these assumptions were not met, the nonparametric Kruskal–Wallis test was used. When a significant main effect was observed, a post-hoc comparison (*t*-test or Mann–Whitney *U* test) was performed to compare variables among clusters. Statistical significance was set at *P* < 0.05 and adjusted using Bonferroni’s correction. All statistical analyses were performed using R (version 4.3.0). In addition, to validate the normal locomotion model, we used the measured gait data reported by Bovi et. al. [40]. The data included mean values and standard deviations for joint angles and muscle activities during one gait cycle in 20 healthy adults.

## Results

### Generation of normal locomotion

**Fig. 2:**
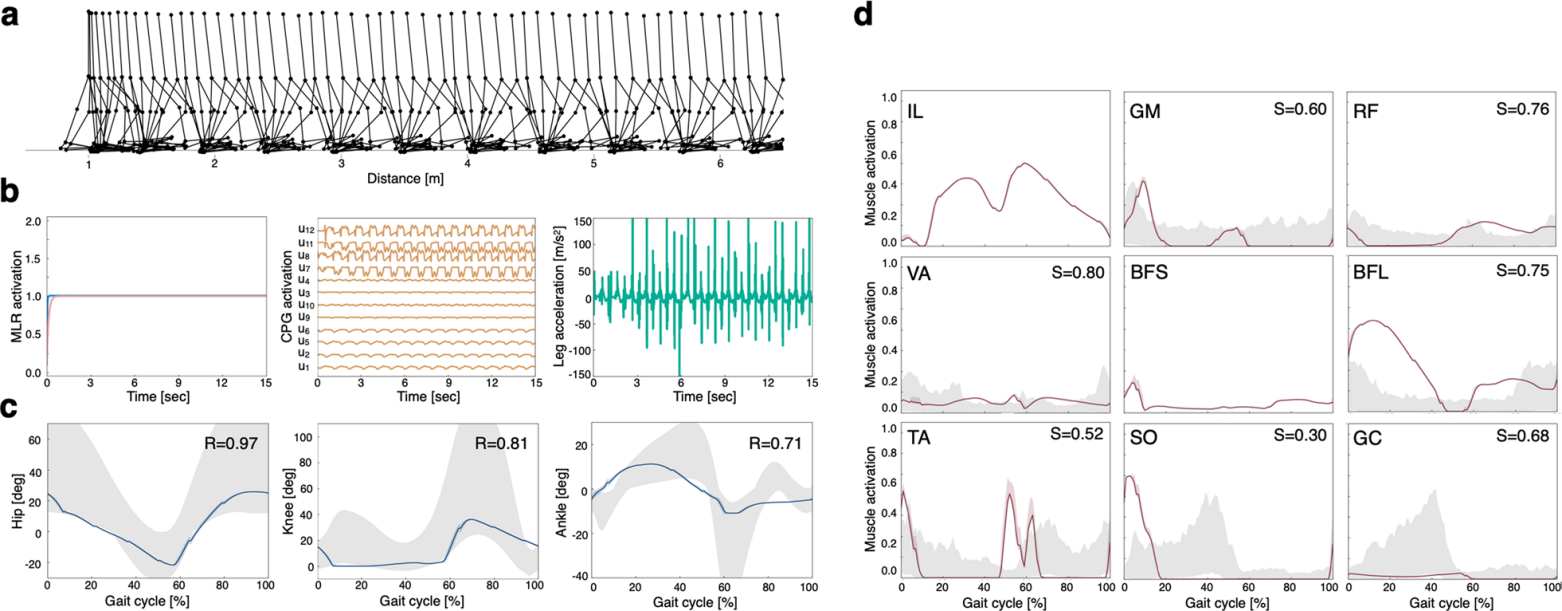
Simulation results of the normal locomotion model. **a** Stick diagram of the normal model. **b** Midbrain locomotor region (MLR) activation, central pattern generator (CPG) activation, and leg acceleration during 15 s of walking. **c** Joint angles. **d** Muscle activations. A gait cycle is the period of events during locomotion from the time one foot contacts the ground until the same foot contacts the ground again. The solid lines represent the mean of the five gait cycles (steps 4 to 8) of the left leg in one simulation, and the shaded region represents the standard deviation (SD). The gray areas represent measured data [40] (μ ± 2 SD). *R* is the correlation coefficient, and *S* represents cosine similarity

After 2000 generations of the GA, the normal model (Fig. 1) achieved a stable gait (Fig. 2a). The gait pattern qualitatively mimicked human bipedal locomotion. The PPN and CnF neurons in the MLR model immediately reached a steady-state value of 1.0 during walking (MLR model parameters: ${s}^{\text{PPN}}$=1.0, ${s}^{\text{CnF}}$=1.0), and periodic waveforms were observed in the CPG activations and right lower leg anterior–posterior acceleration (Fig. 2b). These results indicate that this model had a steady-state normal gait. Figure 2c and d show the joint angles and muscle activation, respectively. To validate the simulation results, we calculated the correlation coefficient (*R*) and cosine similarity (*S*) between the simulation and measured data [40]. The IL and BFS of the muscle activations were not compared with the simulation results because of the lack of measurement data [40]. IL activity is present mainly in the middle and BFS activity at the beginning and end of the gait cycle, which was qualitatively comparable to our simulation results [41]. Thus, the gait of the normal model was qualitatively and quantitatively similar to those reported in the previous studies [25, 26].

### Gait patterns under abnormal conditions

**Fig. 3:**
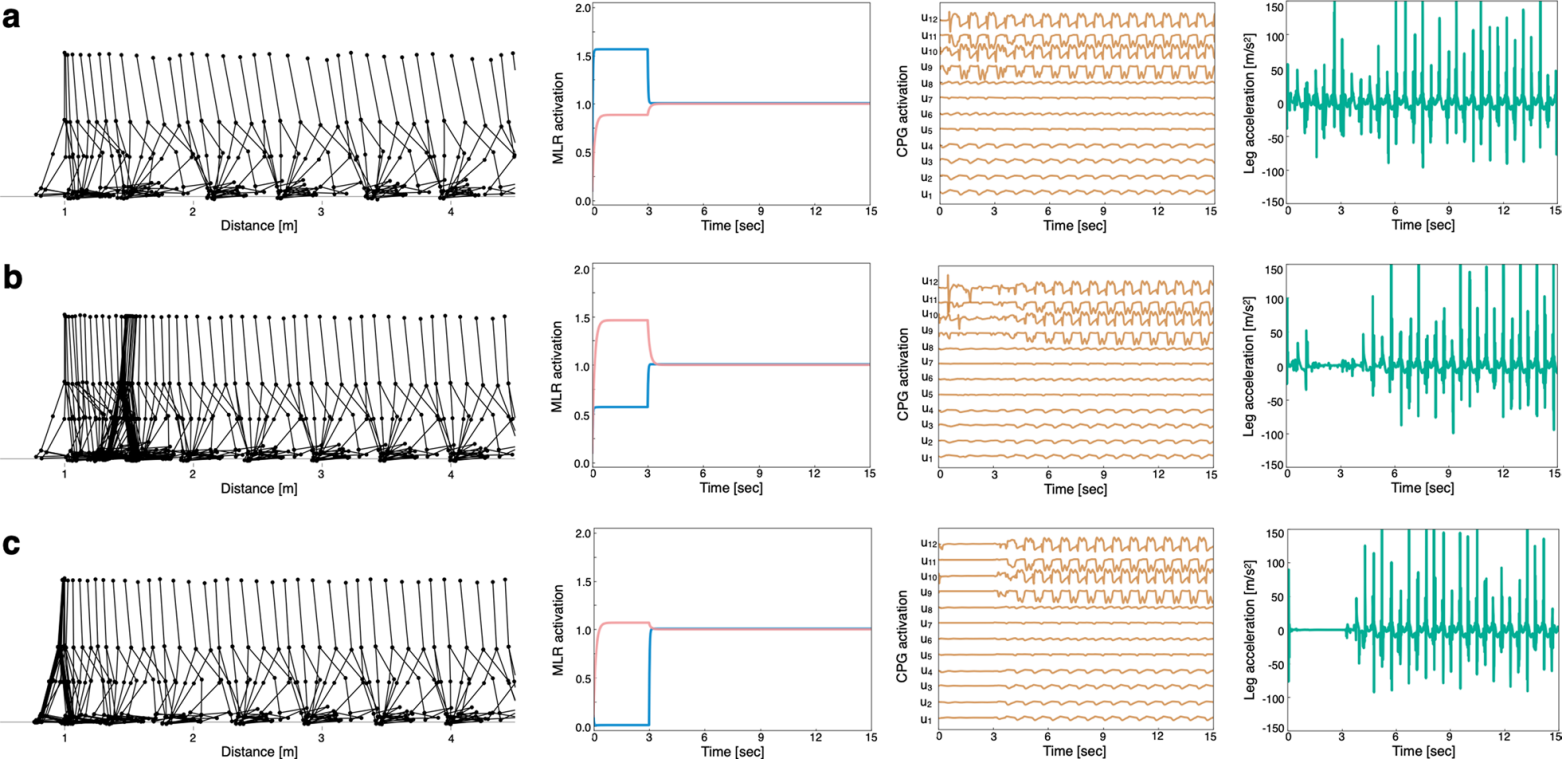
Three typical gait patterns under abnormal conditions. **a** ${s}^{\text{PPN}}$ = 0.84, ${s}^{\text{CnF}}$ = 1.56. **b** ${s}^{{\text{PP}}{\text{N}}}$ = 1.52, ${s}^{\text{CnF}}$ = 0.56. **c** ${s}^{\text{PPN}}$ = 1.14, ${s}^{\text{CnF}}$ = 0.00. Columns from left to right show a stick diagram of the model, midbrain locomotor stimulation (MLR) activation, central pattern generator (CPG) activation, and leg acceleration. Red and blue in column 2 indicate pedunculopontine nucleus (PPN) and cuneiform nucleus (CnF) activities, respectively

The values of ${s}^{\text{PPN}}$ and ${s}^{\text{CnF}}$ were permutatively varied from 0.00 to 2.00 in 0.01 increments only during the initial 3 s of walking. Among the simulation results obtained with these 40,000 parameter sets, 3,184 models walked without falling. Figure 3 shows three representative gait simulations. Figure 3a illustrates a case without marked variation in CPG activation or leg acceleration, indicating a steady gait. Figure 3b and c illustrate cases with low CPG activation and leg acceleration activity at the beginning of walking, implying a freeze-like gait. Thus, we observed different gait patterns for different parameters.

### Characteristics of the clusters

**Fig. 4:**
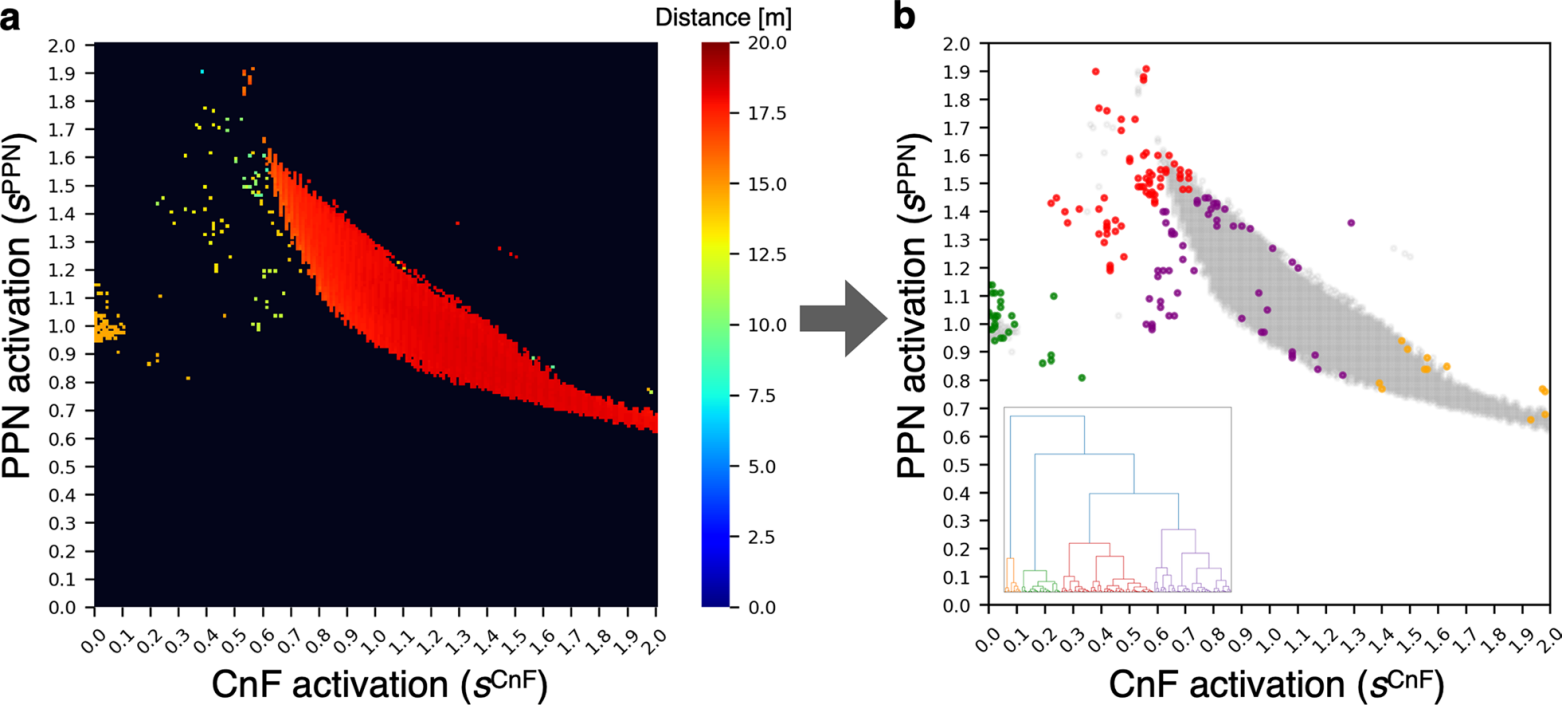
Heat map based on walking distance and identified clusters. **a** Based on the values of the ${s}^{\text{PPN}}$ and ${s}^{\text{CnF}}$ parameters, walking distances over 15 s are indicated by color changes. **b** FOG instances were determined using the FOG-identifying algorithm [17] followed by hierarchical cluster analysis. Clusters 1–4 are shown in green, red, purple, and yellow, respectively. Gray indicates parameter combinations, whose results were not identified as FOGs. PPN, pedunculopontine nucleus; CnF, cuneiform nucleus

We created a heat map based on walking distance to visually understand the differences in gait (Fig. 4a). Subsequently, the FOG-identifying algorithm [17] was used to determine FOG instances. As shown in Fig. 4b, the red area around the normal gait (model parameters: ${s}^{\text{PPN}}$=1.0, ${s}^{\text{CnF}}$=1.0) disappeared, and parameter combinations that were not identified as FOGs turned gray. Finally, HCA based on ${s}^{\text{PPN}}$ and ${s}^{\text{CnF}}$ was applied to the 156 models identified as FOG. A large increase in the agglomeration coefficient reduction rate was observed between clusters 3 and 4 (57.46%), followed by an increase of 28.21% between clusters 4 and 5 and 25.31% between clusters 5 and 6, indicating a plateau in the reduction rate. Therefore, we set the number of clusters to four. This result was verified by visual inspection of the dendrogram (Fig. 4b). Clusters 1 (green), 2 (red), 3 (purple), and 4 (yellow) contained 27, 64, 53, and 12 models, respectively (see Additional file 2).

### Comparison of FOG characteristics among clusters

**Fig. 5:**
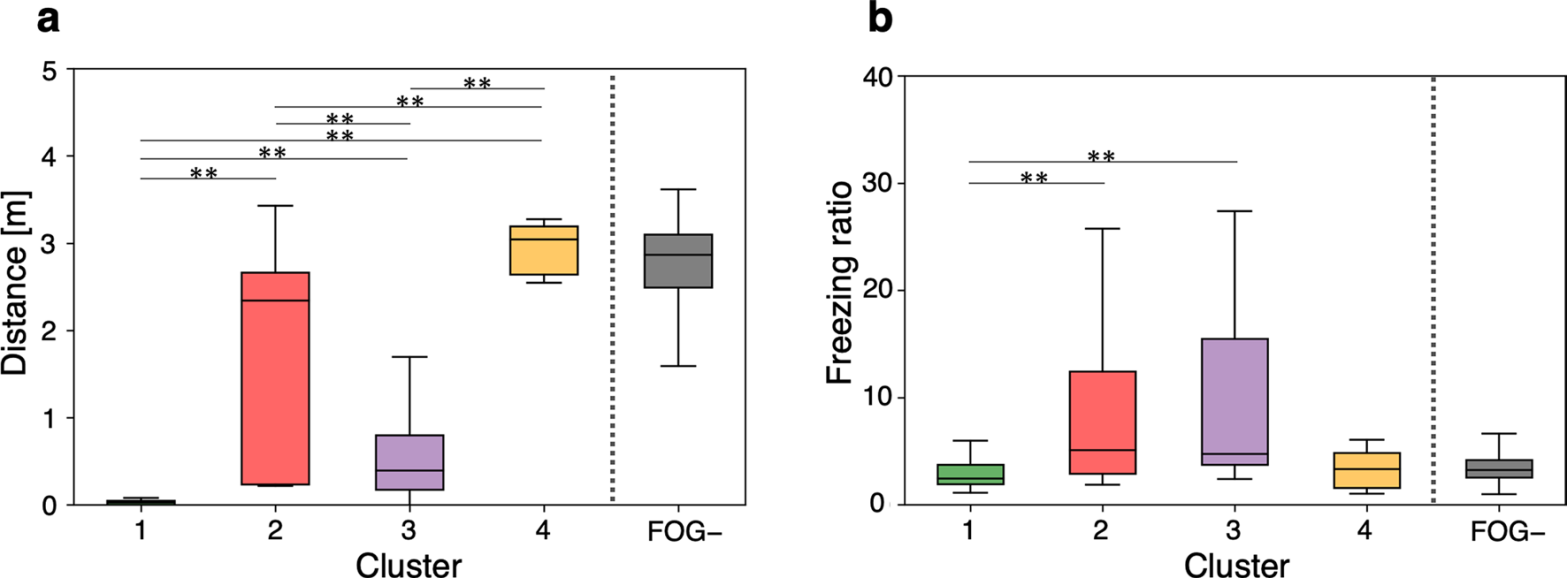
Differences in FOG characteristics among clusters. The central line of the boxplot indicates the median value, and upper and lower hinges indicate the first and third quartiles of data, respectively. Clusters 1–4 are shown in green (*n* = 27), red (*n* = 64), purple (*n* = 53), and yellow (*n* = 12), respectively. FOG- is the group of instances not identified as FOG by identifying the algorithm. Asterisks represent significant differences (^∗^*P* < 0.05, ^∗∗^*P* < 0.01 Mann–Whitney *U* test with Bonferroni’s correction). **a** Walking distances during the initial 3 s of walking (Kruskal–Wallis test: *P* < 0.001; Mann–Whitney *U* test with Bonferroni’s correction: *P* < 0.001 for cluster 1 vs. cluster 2, *P* < 0.001 for cluster 1 vs. cluster 3, *P* < 0.001 for cluster 1 vs. cluster 4, *P* < 0.001 for cluster 2 vs. cluster 3, *P* < 0.001 for cluster 2 vs. cluster 4, *P* < 0.001 for cluster 3 vs. cluster 4). **b** Freezing ratio during the initial 3 s of walking (Kruskal–Wallis test: *P* < 0.001; Mann–Whitney *U* test with Bonferroni’s correction: *P* < 0.001 for cluster 1 vs. cluster 2, *P* < 0.001 for cluster 1 vs. cluster 3, *P* ≥ 1.00 for cluster 1 vs. cluster 4, *P* ≥ 1.00 for cluster 2 vs. cluster 3, *P* = 0.070 for cluster 2 vs. cluster 4, *P* = 0.187 for cluster 3 vs. cluster 4)

Based on the clinical classification [19, 20], we compared the walking distance and freezing ratio during the initial 3 s of walking among the clusters (Fig. 5). As shown in Fig. 5a, walking distances were the shortest in cluster 1 and longest in cluster 4. Walking distances significantly differed among clusters. As shown in Fig. 5b, the freezing ratio was lower in cluster 1 and higher in clusters 2 and 3. Thus, cluster 1 exhibited little forward movement and leg motion during FOG, whereas cluster 4 exhibited a gait similar to that of the FOG-negative model. Clusters 2 and 3 exhibited FOG intermediate to that of clusters 1 and 4. Time series plots of walking distance, leg acceleration, joint angles, and muscle activities in these clusters are presented in Additional file 1.

## Discussion

**Fig. 6:**
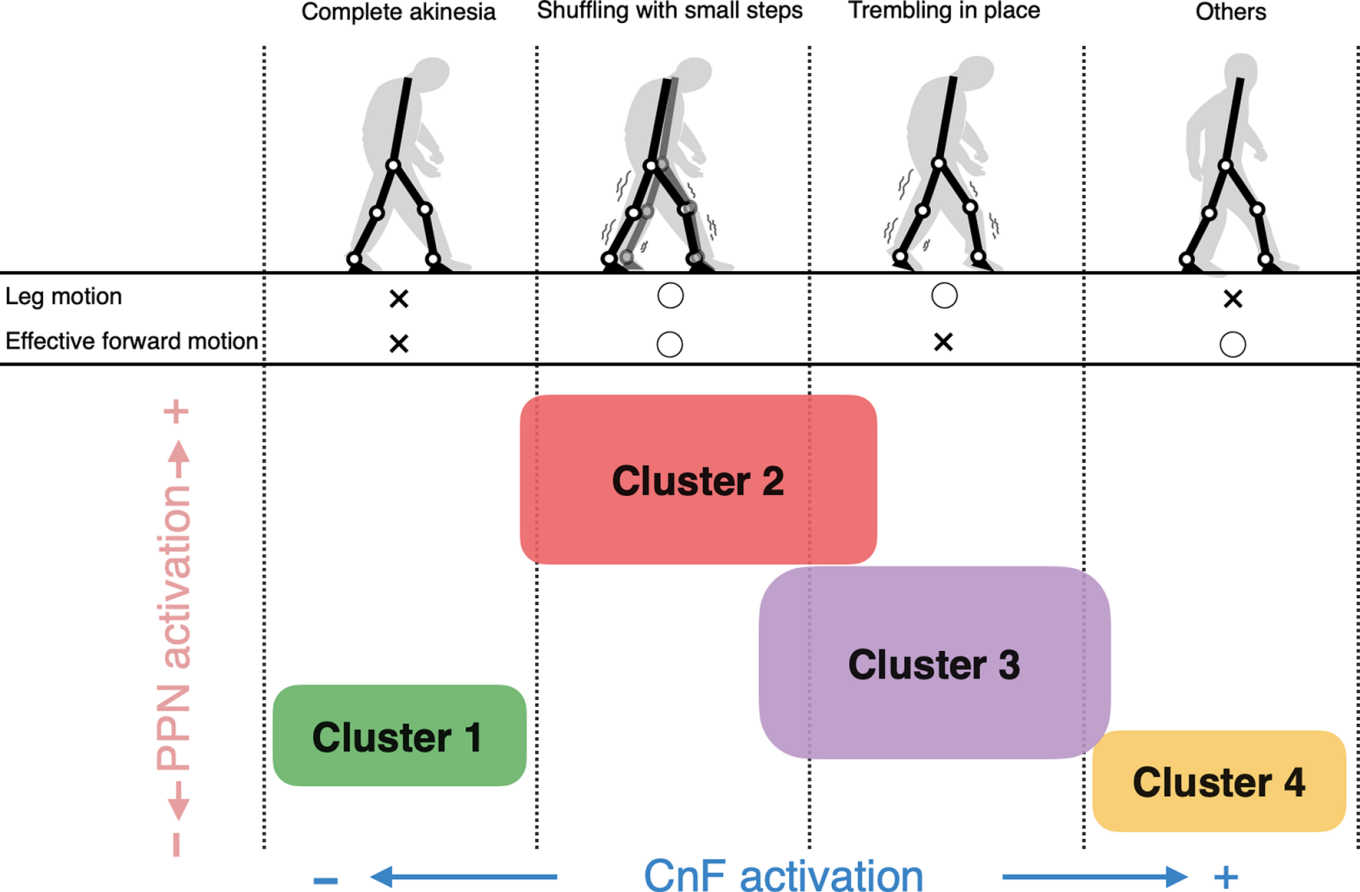
Hypothetical scheme for categorizing clusters and FOG types. Each cluster based on pedunculopontine nucleus (PPN) and cuneiform nucleus (CnF) parameters was mapped to the clinical classification of FOG [19, 20], according to differences in FOG characteristics (Fig. 3). Leg motion and effective forward motion correspond to the freezing ratio and walking distance during the first 3 s of walking, respectively. Complete akinesia: no observable motion of the legs, shuffling with small steps: FOG associated with very small shuffling steps and with minimal forward movement, trembling in place: FOG with some leg trembling but no effective forward motion

We used the gait model consisting of a body with seven links and 18 muscles as well as a neural system with MLR and CPG controllers and confirmed to walk successfully following the fitting of the internal parameters using the GA. We investigated whether FOG could be observed under conditions of abnormal brainstem activity by modifying the parameters of the PPN and CnF models during only the initial 3 s of walking. An identification algorithm [17] was used to test for the presence of FOG among the 40,000 simulated parameter sets. HCA was carried out on the identified 156 instances, and four clusters were identified. Comparisons of physical movements during FOG revealed differences among the clusters. Our results present evidence that modifications in PPN and CnF activities may be linked with the pathogenesis of FOG and its subtypes (Fig. 6), providing potential objective explanations for the qualitative clinical classification of FOG [19, 20].

### Applicability of simulation

The FOG simulations in this study were based on reported physiological findings, indicating the validity of replicating real-world scenarios in this type of model. Caggiano et al. [7], Josset et al. [8], and Dautan et al. [9] showed that changes in CnF and PPN activities modulate differences in walking speed and locomotor patterns in mice. However, designing appropriate animal models to investigate FOG is difficult because of its dependence on intentional locomotor activity and environmental responses [12]. Computer simulations, such as those employed in the present study, which incorporate experimental findings, are thus an effective means of investigating FOG. As the FOG-identifying algorithm is also used in clinical settings [17], our approach facilitates comparisons between models and patient reports as well as validation of the model outputs.

### Differences between subtypes in the FOG model

Functionally different PPN and CnF activities may affect each other, resulting in the appearance of FOG subtypes. In Fig. 6, cluster 1 was assumed to represent complete akinesia because both leg motion and effective forward motion were rarely observed. These observed behaviors were caused by a remarkable weakening in CnF activity and a consequent decrease in CPG activity. The simulation results support a study protocol design of deep brain stimulation (DBS) of the CnF [42] and indicate that the occurrence of FOG was decreased by stimulating the spinal cord with CPG [43]. Both PPN and CnF activities were higher in clusters 2 and 3 than in cluster 1. Cluster 2 showed high PPN activity, correlated with lower muscle tone, and consequently effective forward motion was observed. This cluster was therefore assumed to represent shuffling with small steps. Cluster 3 showed lower PPN activity than cluster 2, correlated with a higher muscle tone, and consequently effective forward was limited. This cluster was therefore assumed to represent trembling in place. However, the boundary between clusters 2 and 3 was vague (Fig. 4). This attribution is supported by the findings of a previous study wherein shuffling with small steps was difficult to separate from trembling in place, even when observed by experts [20].

### Implications for FOG interventions

Treatment of FOG requires methods tailored to its variations. Although L-dopa, a medication used to treat PD, is used for treating FOG, its efficacy has been inconsistent [18], as has the application of visual and auditory cue stimulations [44, 45]. The results of the present study suggest that these unclear intervention effects may be explained by the anatomical and functional differences between the CnF and PPN. The CnF has several connections to the midbrain, which is believed to receive visual and auditory information. In contrast, the PPN has many connections to the basal ganglia [7, 9], which likely enable its susceptibility to medications such as L-dopa [46]. Based on these findings, the modulation of CnF and PPN activities may be effective for treating FOG depending on the specific type, as shown in Fig. 6. For example, for instances connected to cluster 2, intervention with cue stimulation may be more effective than L-dopa administration.

Although DBS is an effective therapy for PD [47], its efficacy for FOG has been inconsistent [48]. The DBS in FOG treatment often targets the MLR, especially around the PPN. The PPN DBS provides benefits in postural stability and fall prevention [49, 50]; however, its effects are limited and may be reduced, especially when stimulating nerves with advanced degeneration [51]. In contrast, Goetz et al. (2019) reported that electrode placement in the CnF, or in a region bordering it, was associated with improved FOG in DBS [52]. These findings indicate that effective DBS stimulation areas may differ depending on the subtype or symptom of FOG. As shown in Fig. 6, the results of this study may provide insights relevant for selecting stimulation areas for specific FOG subtypes.

### Limitations and future work

The simplified skeletal model used in this study enabled us to gain a comprehensive understanding of the pathophysiology. However, the model was limited to two dimensions, and the neural models were mathematically abstracted without explicitly including the cerebral cortical and subcortical functions*.*

In the skeletal model, the number of joints and segments were limited and did not explicitly include the arms. Arm swings during gait contribute to the dynamic balance [53], and therefore our model could not probably simulate recovery from postural instability. In addition, although reduced arm swing during gait was reported in PD [54], no significant difference in arm swing with or without FOG was noted [55]. Our model could be useful if limited to observing FOG features while walking on flat surfaces. In the muscle model, we only used a certain parameter set for healthy subjects and did not simulate the gait of other populations, such as the elderly [56] or frail [57]. Thus, the model was not adapted to individual physical characteristics. This limited use of parameter sets was due to the focus on simulation of changes in brainstem activity. Consequently, we observed alterations in gait due to changes in neural activity on the computer. In the neural models, it was difficult to analyze differences in spike firing among individual neurons because we used the rate model. In addition, we modeled the brainstem as the MLR, with only the PPN and CnF associated with gait. Our models are not suitable for discussing changes in neural dynamics due to networks connecting multiple brain regions and various ion channels. In particular, the cerebral cortex and subcortical measurement data during FOG [58, 59] are difficult to interpret using only this model. Rather, our model specializes in the minimum necessary neural function of gait by imposing such limitation to represent a closed-loop system in human gait.

Increasing the complexity of the model, for example, by adding more detailed structures [26, 60] or higher-level neural models [33, 61], would be a reasonable step to improve our findings. We plan to measure large amounts of FOG event data and investigate more detailed and individualized FOG instances by combining large-scale models with actual patient data. Several clinical issues, such as those regarding the conditions under which FOG event is more likely to occur or its change over time, need to be resolved.

## Conclusions

We investigated the pathogenesis of FOG and its subtypes using a two-dimensional neuromusculoskeletal model. In the simulation, the parameters of the PPN and CnF models in the brainstem were modified during the initial 3 s of walking, resulting in instances of FOG comparable to reported observations. A comprehensive examination of 40,000 PPN and CnF parameter sets suggests that the generation of FOG and its subtypes may be due to changes in the activities of these two nuclei. These results suggest insights into the development of rehabilitation methods tailored to each severity and subtype, medication adjustments, and effective rehabilitation devices.

## Supplementary Information

Additional file 1.

Additional file 2.

## Data Availability

All data generated or analyzed during this study are included in this published article and its supplementary information files.

## Abbreviations

CnF: Cuneiform nucleus
CPG: Central pattern generator
FOG: Freezing of gait
GA: Genetic algorithm
HAT: Head, arms, torso
HCA: Hierarchical cluster analysis
IL: Iliopsoas
MLR: Midbrain locomotor region
PD: Parkinson’s disease
PPN: Pedunculopontine nucleus
