# Supplementary material for: Abnormal activity in the brainstem affects gait in a neuromusculoskeletal model
Source: J Neuroeng Rehabil. 2025 Apr 4;22:73. doi: 10.1186/s12984-025-01596-x (PMC11969973; doi:10.1186/s12984-025-01596-x)
Supplement: Supplementary file 1 — Additional file 1. [file 12984_2025_1596_MOESM1_ESM.pdf]

Abnormal activity in the brainstem affects gait in a neuromusculoskeletal model

Parameters for the Musculoskeletal model

We constructed the skeletal and muscle models using the parameters presented in Table A1 and Table A2, respectively.

Parameters for the MLR model

$\tau^{\mathrm{PPN}} = 0.20, \tau^{\mathrm{CnF}} = 0.04, \beta^{\mathrm{PPN}} = 0.60, \beta^{\mathrm{CnF}} = 0.50, w^{\mathrm{HC}} = 1.50.$

The initial values are as follows:

$u^{\mathrm{PPN}} = 0.10, u^{\mathrm{CnF}} = 0.10.$

Parameters for the CPG model

$$\tau_1, \dots, \tau_4 = 0.05, \tau_5 \dots, \tau_{12} = 0.025, \tau_1', \dots, \tau_4' = 0.60, \tau_5', \dots, \tau_{12}' = 0.30, \beta = 2.5, u_0 = 4.043.$$

$$w_{ij}^{\mathrm{CPG}} = \begin{cases} -2.0 & (i,j) \in \{(1,2),(2,1),(3,4),(4,3),(5,6),(6,5),(7,8),(8,7), \\ & \quad (9,10),(10,9),(11,12),(12,11)\}, \\ -1.0 & (i,j) \in \{(1,3),(3,1),(2,4),(4,2),(6,1),(6,2),(8,3),(8,4), \\ & \quad (10,1),(10,2),(12,3),(12,4)\}, \\ 0.0 & \text{otherwise.} \end{cases}$$

Feedback signals from the musculoskeletal system are defined as follows:

$\mathrm{Feed}_1 = w_1^{\mathrm{Feed}} \theta_{Rthigh}^{\mathrm{seg}} - w_2^{\mathrm{Feed}} \theta_{Lthigh}^{\mathrm{seg}} + w_3^{\mathrm{Feed}} \theta_{Rshank}^{\mathrm{seg}} h(\mathrm{GRF}_R) + w_4^{\mathrm{Feed}} h(\mathrm{GRF}_L),$

$\mathrm{Feed}_2 = -w_5^{\mathrm{Feed}} \theta_{Rthigh}^{\mathrm{seg}} + w_6^{\mathrm{Feed}} \theta_{Lthigh}^{\mathrm{seg}} - w_7^{\mathrm{Feed}} \theta_{Rshank}^{\mathrm{seg}} h(\mathrm{GRF}_R) - w_8^{\mathrm{Feed}} h(\mathrm{GRF}_L),$

$\mathrm{Feed}_3 = w_1^{\mathrm{Feed}} \theta_{Lthigh}^{\mathrm{seg}} - w_2^{\mathrm{Feed}} \theta_{Rthigh}^{\mathrm{seg}} + w_3^{\mathrm{Feed}} \theta_{Lshank}^{\mathrm{seg}} h(\mathrm{GRF}_L) + w_4^{\mathrm{Feed}} h(\mathrm{GRF}_R),$

$\mathrm{Feed}_4 = -w_5^{\mathrm{Feed}} \theta_{Lthigh}^{\mathrm{seg}} + w_6^{\mathrm{Feed}} \theta_{Rthigh}^{\mathrm{seg}} - w_7^{\mathrm{Feed}} \theta_{Lshank}^{\mathrm{seg}} h(\mathrm{GRF}_L) - w_8^{\mathrm{Feed}} h(\mathrm{GRF}_R),$

$\mathrm{Feed}_5 = -w_9^{\mathrm{Feed}} \theta_{Lshank}^{\mathrm{seg}} h(\mathrm{GRF}_L),$

$\mathrm{Feed}_6 = w_{10}^{\mathrm{Feed}} \theta_{Lshank}^{\mathrm{seg}} h(\mathrm{GRF}_L),$

$\mathrm{Feed}_7 = -w_9^{\mathrm{Feed}}\theta_{Rshank}^{\mathrm{seg}}h(\mathrm{GRF}_R),$

$\mathrm{Feed}_8 = w_{10}^{\mathrm{Feed}}\theta_{Rshank}^{\mathrm{seg}}h(\mathrm{GRF}_R),$

$\mathrm{Feed}_9 = -w_{11}^{\mathrm{Feed}}\theta_{Rshank}^{\mathrm{seg}}h(\mathrm{GRF}_R) - w_{12}^{\mathrm{Feed}}\theta_{Lshank}^{\mathrm{seg}}h(\mathrm{GRF}_L) - w_{13}^{\mathrm{Feed}}\dot{\theta}_{Rshank}^{\mathrm{seg}}h(\mathrm{GRF}_R),$

$\mathrm{Feed}_{10} = w_{14}^{\mathrm{Feed}}\theta_{Rshank}^{\mathrm{seg}}h(\mathrm{GRF}_R) + w_{15}^{\mathrm{Feed}}\theta_{Lshank}^{\mathrm{seg}}h(\mathrm{GRF}_L) + w_{16}^{\mathrm{Feed}}\dot{\theta}_{Rshank}^{\mathrm{seg}}h(\mathrm{GRF}_R),$

$\mathrm{Feed}_{11} = -w_{11}^{\mathrm{Feed}}\theta_{Lshank}^{\mathrm{seg}}h(\mathrm{GRF}_R) - w_{12}^{\mathrm{Feed}}\theta_{Rshank}^{\mathrm{seg}}h(\mathrm{GRF}_R) - w_{13}^{\mathrm{Feed}}\dot{\theta}_{Lshank}^{\mathrm{seg}}h(\mathrm{GRF}_L),$

$\mathrm{Feed}_{12} = w_{14}^{\mathrm{Feed}}\theta_{Lshank}^{\mathrm{seg}}h(\mathrm{GRF}_R) + w_{15}^{\mathrm{Feed}}\theta_{Rshank}^{\mathrm{seg}}h(\mathrm{GRF}_R) + w_{16}^{\mathrm{Feed}}\dot{\theta}_{Lshank}^{\mathrm{seg}}h(\mathrm{GRF}_L),$

$h(x) = \begin{cases} 0\ (x \le 0), \\ 1\ \ (x > 0). \end{cases}$

$w_1^{\mathrm{Feed}} = 0.749, w_2^{\mathrm{Feed}} = 8.359, w_3^{\mathrm{Feed}} = 1.766, w_4^{\mathrm{Feed}} = 6.547, w_5^{\mathrm{Feed}} = 3.271,$

$w_6^{\mathrm{Feed}} = 0.408, w_7^{\mathrm{Feed}} = 1.044, w_8^{\mathrm{Feed}} = 0.943, w_9^{\mathrm{Feed}} = 4.026, w_{10}^{\mathrm{Feed}} = 8.024,$

$w_{11}^{\mathrm{Feed}} = 11.616, w_{12}^{\mathrm{Feed}} = 4.558, w_{13}^{\mathrm{Feed}} = 1.785, w_{14}^{\mathrm{Feed}} = 8.608, w_{15}^{\mathrm{Feed}} = 2.890,$

$w_{16}^{\mathrm{Feed}} = 43.438.$

Parameters for the α-motor neurons

$w_{Rbfl\,5}^{\alpha} = w_{Lbfl\,7}^{\alpha} = 0.265, w_{Rbfl\,2}^{\alpha} = w_{Lbfl\,4}^{\alpha} = 3.188, w_{Rbfs\,5}^{\alpha} = w_{Lbfs\,7}^{\alpha} = 0.230,$

$w_{Rva\,6}^{\alpha} = w_{Lva\,8}^{\alpha} = 0.001, w_{Ril\,1}^{\alpha} = w_{Lil\,3}^{\alpha} = 1.051, w_{Rgm\,2}^{\alpha} = w_{Lgm\,4}^{\alpha} = 0.450,$

$w_{Rrf\,1}^{\alpha} = w_{Lrf\,3}^{\alpha} = 0.168, w_{Rrf\,6}^{\alpha} = w_{Lrf\,8}^{\alpha} = 0.297, w_{Rta\,9}^{\alpha} = w_{Lta\,11}^{\alpha} = 0.556,$

$w_{Rgc\,10}^{\alpha} = w_{Lgc\,12}^{\alpha} = 0.001, w_{Rso\,10}^{\alpha} = w_{Lso\,12}^{\alpha} = 0.030,$

otherwise $w_{m\,i}^{\alpha} = 0.000.$

Posture control

Posture controls are defined as follows:

$\mathrm{P}_{Rbfl} = w_1^{\mathrm{POS}}\theta_{Rhip} + w_2^{\mathrm{POS}}f(\dot{\theta}_{Rhip}),$

$\mathrm{P}_{Lbfl} = w_1^{\mathrm{POS}}\theta_{Lhip} + w_2^{\mathrm{POS}}f(\dot{\theta}_{Lhip}),$

$\mathrm{P}_{Rbfs} = w_3^{\mathrm{POS}}f(\dot{\theta}_{Rknee}),$

$\mathrm{P}_{Lbfs} = w_3^{\mathrm{POS}} f(\dot{\theta}_{Lknee}),$

$\mathrm{P}_{Rva} = -w_4^{\mathrm{POS}} \theta_{Rknee} - w_5^{\mathrm{POS}} f(\dot{\theta}_{Rknee}) h(\mathrm{GRF}_R) + w_6^{\mathrm{POS}} h(\mathrm{GRF}_R),$

$\mathrm{P}_{Lva} = -w_4^{\mathrm{POS}} \theta_{Lknee} - w_5^{\mathrm{POS}} f(\dot{\theta}_{Lknee}) h(\mathrm{GRF}_L) + w_6^{\mathrm{POS}} h(\mathrm{GRF}_L),$

$\mathrm{P}_{Ril} = -w_7^{\mathrm{POS}} \theta_{HAT}^{\mathrm{seg}} - w_8^{\mathrm{POS}} \dot{\theta}_{HAT}^{\mathrm{seg}} h(\mathrm{GRF}_R) - w_9^{\mathrm{POS}} f(\theta_{Rhip}) - w_{10}^{\mathrm{POS}} f(\dot{\theta}_{Rhip}),$

$\mathrm{P}_{Lil} = -w_7^{\mathrm{POS}} \theta_{HAT}^{\mathrm{seg}} - w_8^{\mathrm{POS}} \dot{\theta}_{HAT}^{\mathrm{seg}} h(\mathrm{GRF}_L) - w_9^{\mathrm{POS}} f(\theta_{Lhip}) - w_{10}^{\mathrm{POS}} f(\dot{\theta}_{Lhip}),$

$\mathrm{P}_{Rgm} = w_{11}^{\mathrm{POS}} \theta_{HAT}^{\mathrm{seg}} + w_{12}^{\mathrm{POS}} \dot{\theta}_{HAT}^{\mathrm{seg}} h(\mathrm{GRF}_R) + w_{13}^{\mathrm{POS}} h(\mathrm{GRF}_R),$

$\mathrm{P}_{Lgm} = w_{11}^{\mathrm{POS}} \theta_{HAT}^{\mathrm{seg}} + w_{12}^{\mathrm{POS}} \dot{\theta}_{HAT}^{\mathrm{seg}} h(\mathrm{GRF}_L) + w_{13}^{\mathrm{POS}} h(\mathrm{GRF}_L),$

$\mathrm{P}_{Rrf} = -w_{14}^{\mathrm{POS}} f(\theta_{Rknee}) - w_{15}^{\mathrm{POS}} f(\theta_{Rhip}) h(\mathrm{GRF}_R) + w_{16}^{\mathrm{POS}} h(\mathrm{GRF}_R),$

$\mathrm{P}_{Lrf} = -w_{14}^{\mathrm{POS}} f(\theta_{Lknee}) - w_{15}^{\mathrm{POS}} f(\theta_{Lhip}) h(\mathrm{GRF}_L) + w_{16}^{\mathrm{POS}} h(\mathrm{GRF}_L),$

$\mathrm{P}_{Rta} = w_{17}^{\mathrm{POS}} \dot{\theta}_{Rfoot}^{\mathrm{seg}} h(\mathrm{GRF}_L),$

$\mathrm{P}_{Lta} = w_{17}^{\mathrm{POS}} \dot{\theta}_{Lfoot}^{\mathrm{seg}} h(\mathrm{GRF}_R),$

$\mathrm{P}_{Rgc} = w_{18}^{\mathrm{POS}} f(\theta_{Rankle}) h(\mathrm{GRF}_R),$

$\mathrm{P}_{Lgc} = w_{18}^{\mathrm{POS}} f(\theta_{Lankle}) h(\mathrm{GRF}_L),$

$\mathrm{P}_{Rso} = -w_{19}^{\mathrm{POS}} f(\theta_{Rfoot}^{\mathrm{seg}}) h(\mathrm{GRF}_R) - w_{20}^{\mathrm{POS}} \dot{\theta}_{Rfoot}^{\mathrm{seg}} h(\mathrm{GRF}_R) + w_{21}^{\mathrm{POS}} \theta_{Rankle} h(\mathrm{GRF}_R),$

$\mathrm{P}_{Lso} = -w_{19}^{\mathrm{POS}} f(\theta_{Lfoot}^{\mathrm{seg}}) h(\mathrm{GRF}_L) - w_{20}^{\mathrm{POS}} \dot{\theta}_{Lfoot}^{\mathrm{seg}} h(\mathrm{GRF}_L) + w_{21}^{\mathrm{POS}} \theta_{Lankle} h(\mathrm{GRF}_L),$

$f = \max(0, x).$

$w_1^{\mathrm{POS}} = 3.871, w_2^{\mathrm{POS}} = 0.502, w_3^{\mathrm{POS}} = 0.561, w_4^{\mathrm{POS}} = 1.850, w_5^{\mathrm{POS}} = 1.824,$

$w_6^{\mathrm{POS}} = 0.578, w_7^{\mathrm{POS}} = 29.488, w_8^{\mathrm{POS}} = 22.879, w_9^{\mathrm{POS}} = 0.452, w_{10}^{\mathrm{POS}} = 0.658,$

$w_{11}^{\mathrm{POS}} = 7.598, w_{12}^{\mathrm{POS}} = 10.372, w_{13}^{\mathrm{POS}} = 0.529, w_{14}^{\mathrm{POS}} = 1.434, w_{15}^{\mathrm{POS}} = 1.558,$

$w_{16}^{\mathrm{POS}} = 0.033, w_{17}^{\mathrm{POS}} = 6.385, w_{18}^{\mathrm{POS}} = 1.084, w_{19}^{\mathrm{POS}} = 46.556, w_{20}^{\mathrm{POS}} = 6.083,$

$w_{21}^{\mathrm{POS}} = 1.063.$

Physical responses in posture controls

We confirmed the physical responses in pure postural control without input from the CPG

model (Figure A1). s^{PPN} was set to 1.0, and the HAT was pushed forward and backward at 3N for one second each. The model maintained a standing posture without falling during this manipulation. We observed specific muscle activities that depended on the push direction.

Additional figures for the results

We have described 2D plots of walking distance and left leg acceleration over time in clusters (Figure A2), and time series plot of joint angles and muscle activities during gait (Figures A3-A7).

References

1. Jo S, Massaquoi SG. A model of cerebrocerebello-spinomuscular interaction in the sagittal control of human walking. Biol Cybern. 2007;96:279-307.
2. Ogihara N, Yamazaki N. Generation of human bipedal locomotion by a bio-mimetic neuro-musculo-skeletal model. Biol Cybern. 2001;84:1-11.
3. Aoi S, Ogihara N, Sugimoto Y, Tsuchiya K. Simulating Adaptive Human Bipedal Locomotion Based on Phase Resetting Using Foot-Contact Information. Advanced Robotics, 2008;22:1697-1713.

Table A1. Skeletal model parameters.

Segment	Length [m]	Mass [kg]	Center of gravity [m]	Moment of inertia [kg m^2]
HAT	0.800	44.07	0.3995	5.823
Thigh	0.416	6.50	0.18037	0.117
Shank	0.418	3.055	0.18105	0.048
Foot	0.258 (length), 0.066 (height)	0.975	0.03723	9.347e-5

Note: HAT, head, arms, and torso. Center of gravity is represented as a segment length from the proximal end. These parameters were previously used by Jo and Massaquoi [1].

Table A2. Muscle model parameters.

Muscle	$\bar{F}_m^{\mathrm{CE}}$ [N]	$\bar{L}_m$ [m]	c_m^{PD} [N s/m]	k_m^{PE}	Moment arm [m]
IL	1100	0.35	275	5.85	0.132 (H)
GM	1100	0.30	275	9.10	0.092 (H)
VA	7300	0.26	300	6.80	0.040 (K)
BFS	1000	0.29	200	1.60	0.049 (K)
TA	1650	0.30	200	1.30	0.023 (A)
SO	2150	0.35	200	6.50	0.036 (A)
RF	1800	0.48	300	5.40	0.049 (H) 0.025 (K)
BFL	2750	0.46	275	4.10	0.054 (H) 0.049 (K)
GC	1150	0.56	275	8.25	0.050 (K) 0.040 (A)

Note: IL, iliopsoas, GM gluteus maximus; VA vastus, BFS biceps femoris short head; TA, tibialis anterior; SO, soleus; RF, rectus femoris; BFL, biceps femoris long head; GC, gastrocnemius. Moment arms are those around hip (H), knee (K), or ankle (A) joint. These parameters were previously used by Ogihara and Yamazaki [2] and Aoi et al. [3].

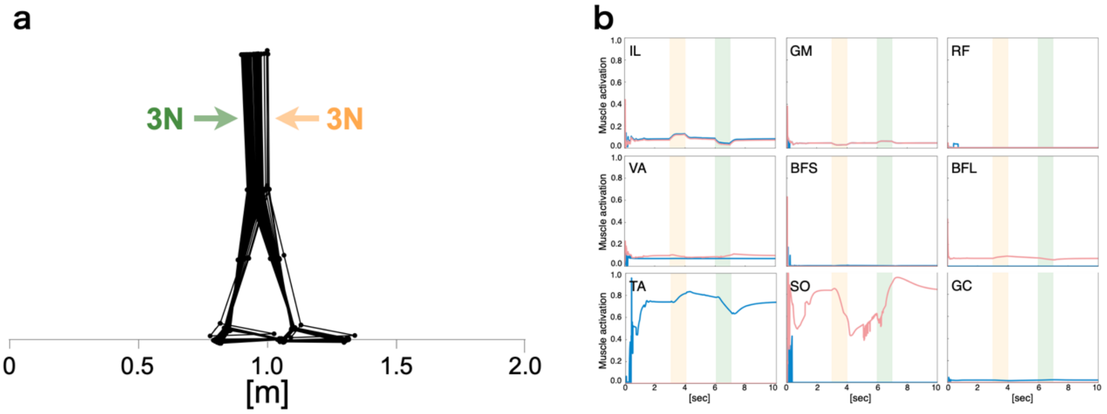

Figure A1. Posture controls for push without input from the central pattern generator (CPG) model. a) maintaining a standing position for 10 s in response to disturbance. **b)** Muscle activities while maintaining a standing position. Orange and green blocks indicate 3N pushes of 1 s duration from forward to backward and from backward to forward, respectively.

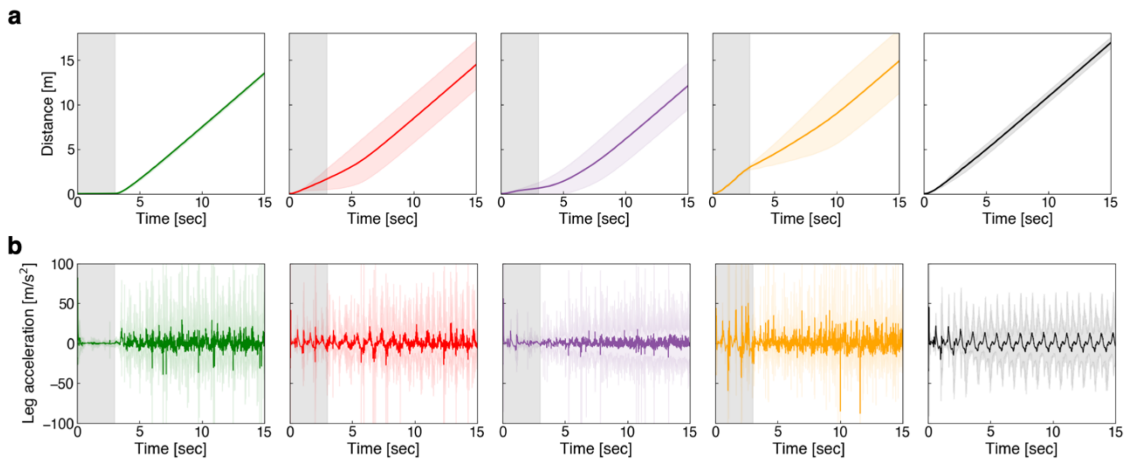

Figure A2. 2D plot of walking distance and left leg acceleration over time. Clusters 1–4 are shown in green, red, purple, and yellow, respectively. The black color represents the FOG negative. The shaded region represents the standard deviation. Gray areas represent modifications to MLR model activities. **a)** 2D plot of walking distance. **b)** 2D plot of left leg anterior–posterior acceleration.

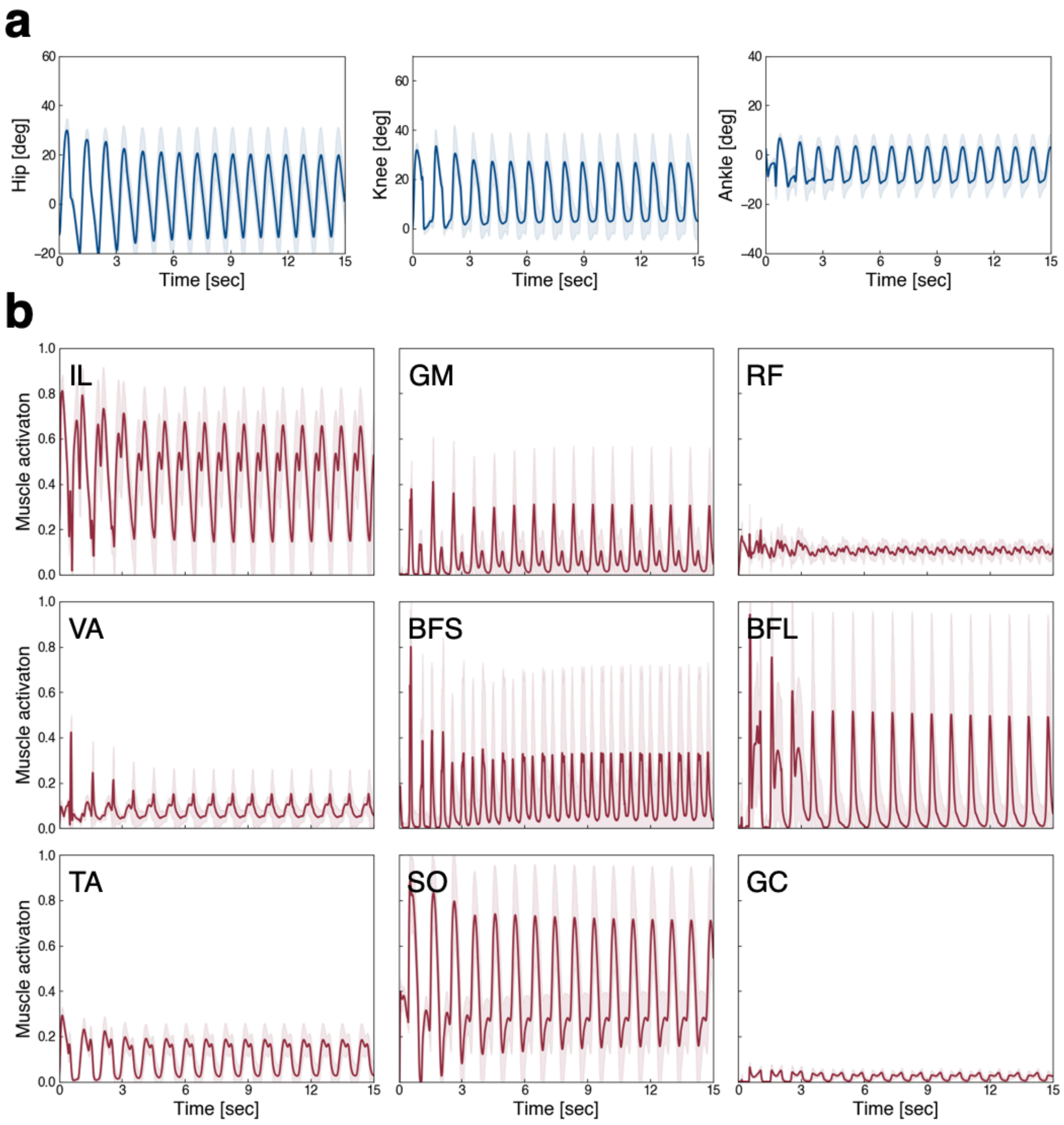

Figure A3. Time series plot of joint angles and muscle activities for the normal locomotion model. The shaded region represents the standard deviation. **a)** Time series plot of joint angles. **b)** Time series plot of muscle activities.

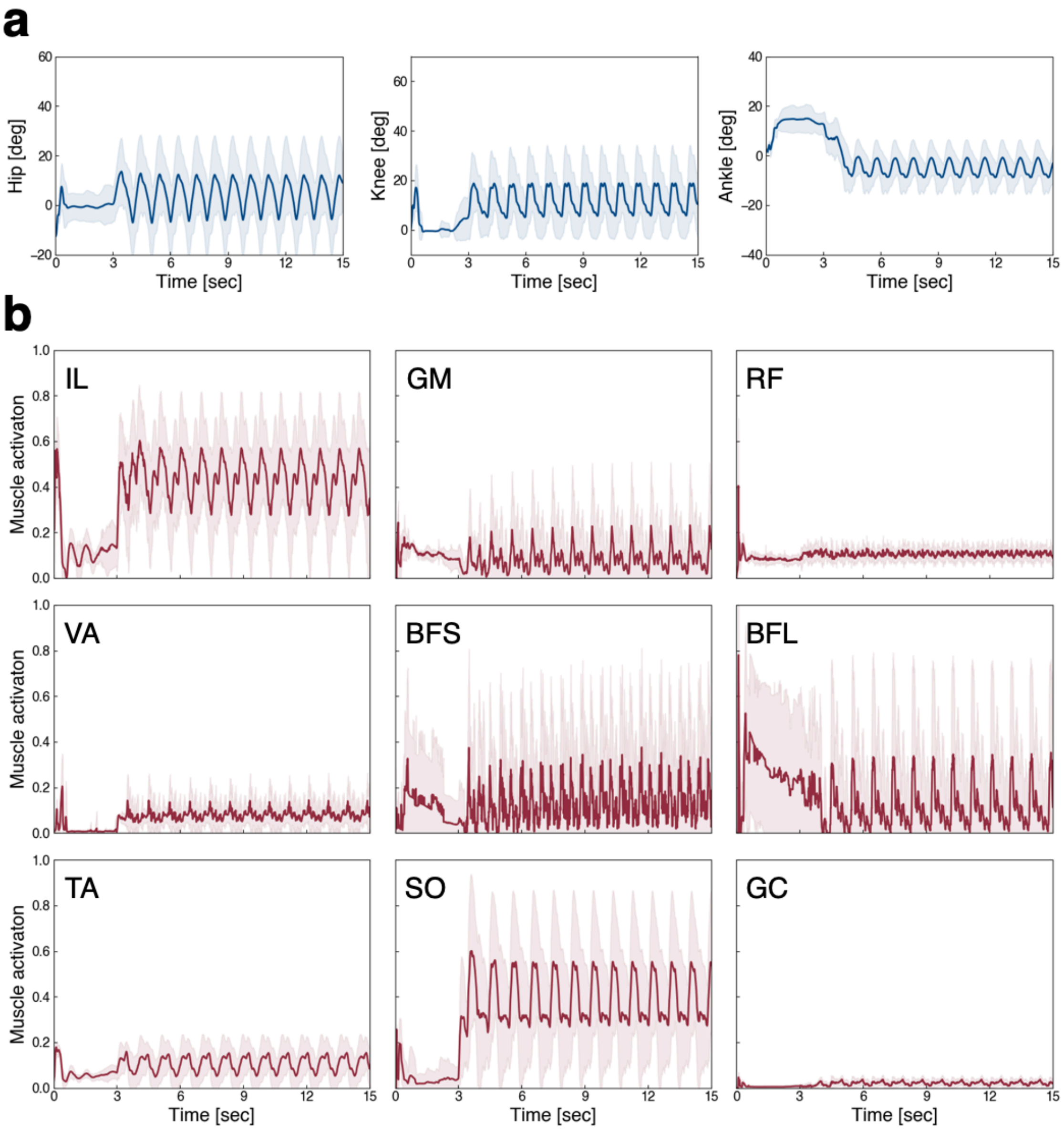

Figure A4. Time series plot of joint angles and muscle activities for Cluster 1. The shaded region represents the standard deviation. **a)** Time series plot of joint angles. **b)** Time series plot of muscle activities.

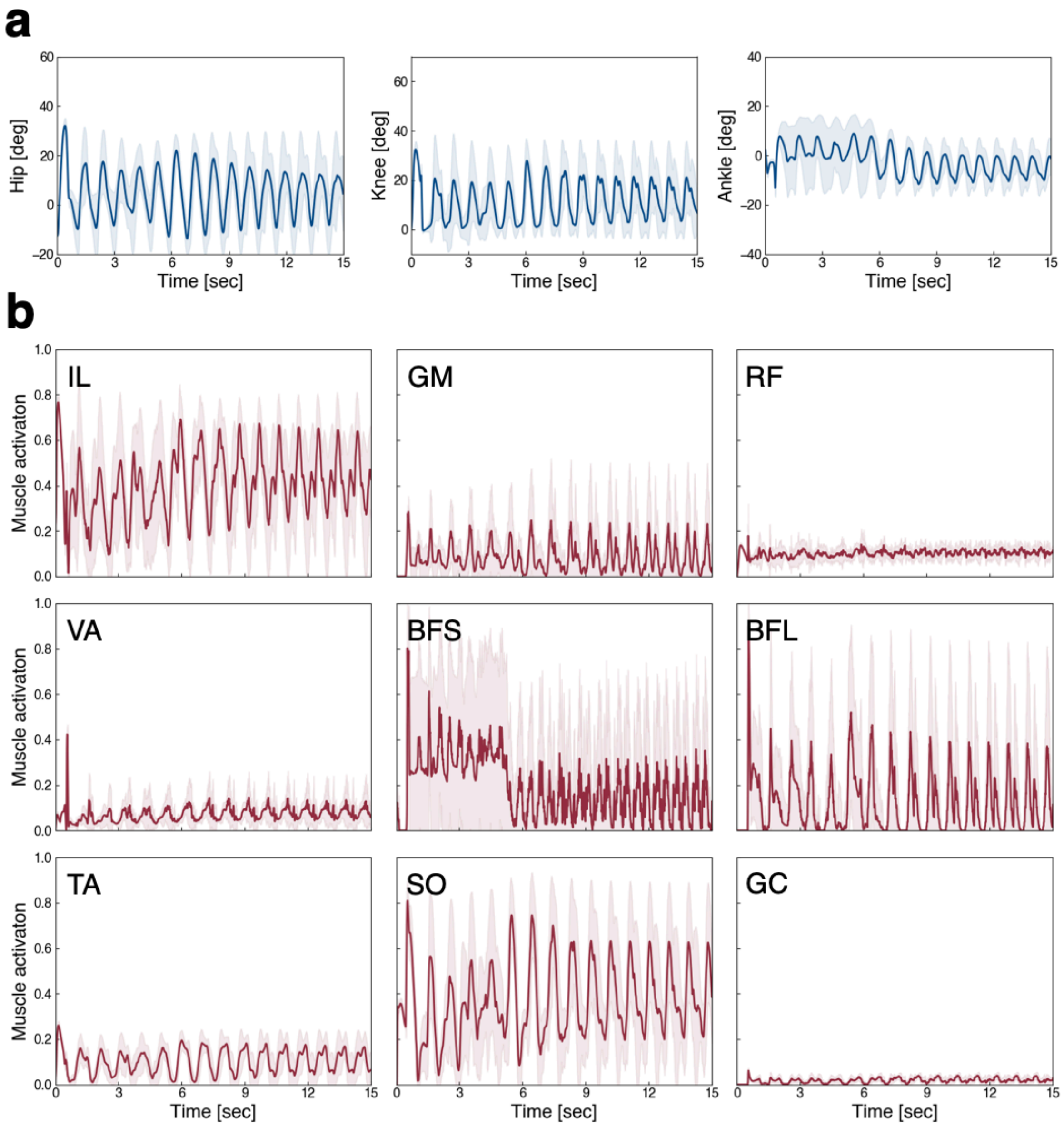

Figure A5. Time series plot of joint angles and muscle activities for Cluster 2. The shaded region represents the standard deviation. **a)** Time series plot of joint angles. **b)** Time series plot of muscle activities.

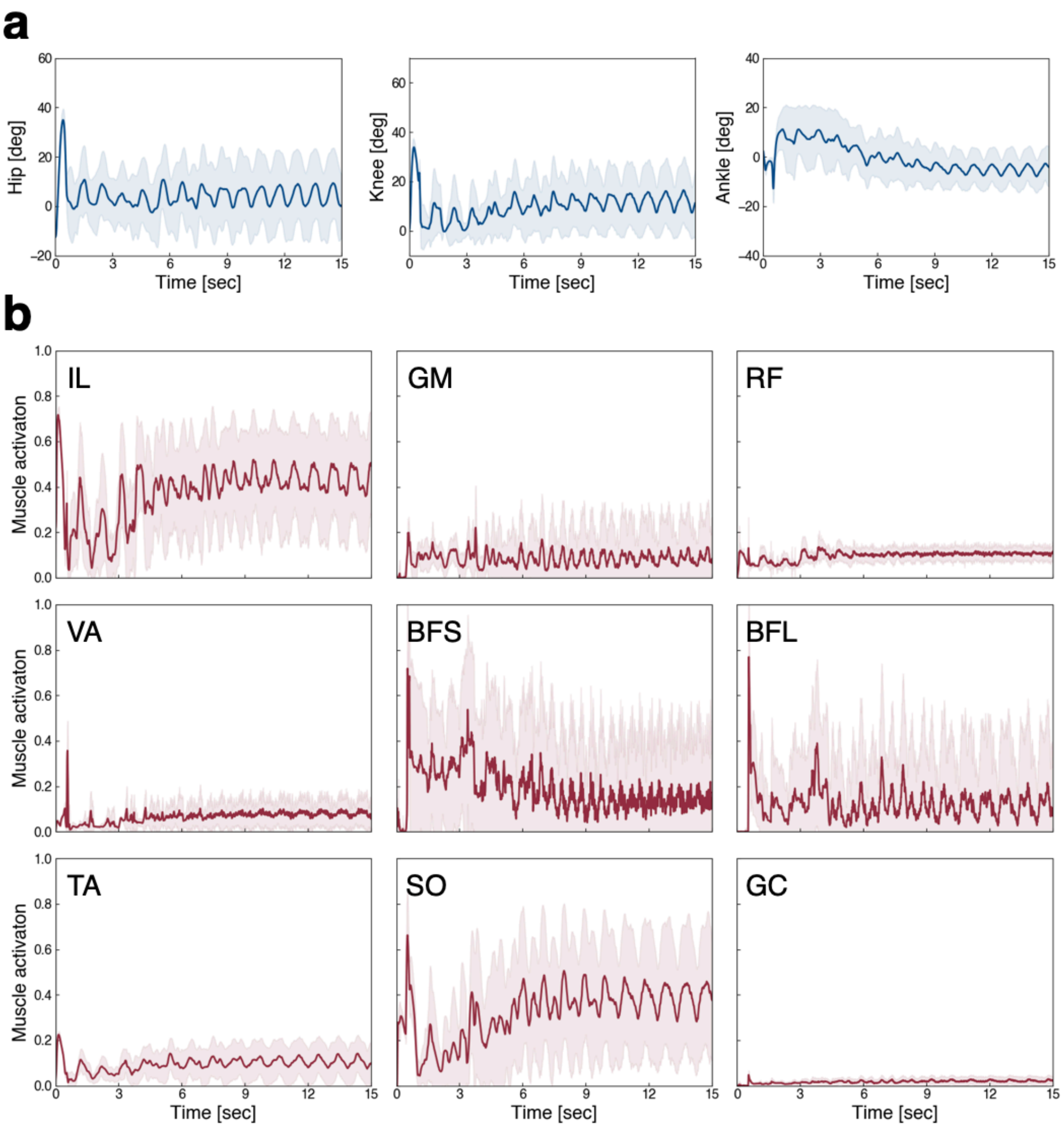

Figure A6. Time series plot of joint angles and muscle activities for Cluster 3. The shaded region represents the standard deviation. **a)** Time series plot of joint angles. **b)** Time series plot of muscle activities.

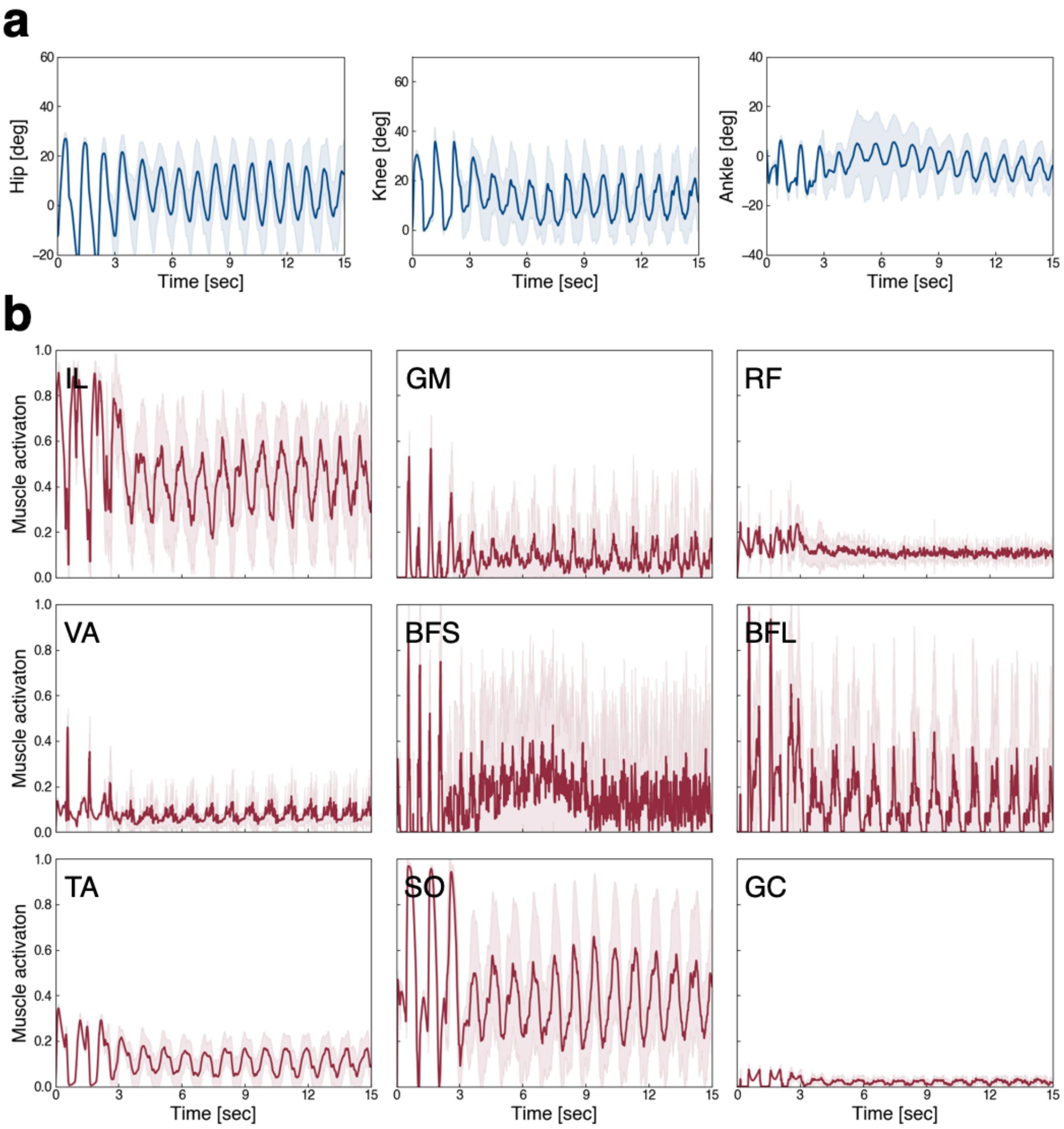

Figure A7. Time series plot of joint angles and muscle activities for Cluster 4. The shaded region represents the standard deviation. **a)** Time series plot of joint angles. **b)** Time series plot of muscle activities.
